# Evolving influence of mental health stigma in Ontario public safety organizations: a qualitative study

**DOI:** 10.24095/hpcdp.46.4.02

**Published:** 2026-04

**Authors:** Leslie Vesely, Cameron A. Mustard, Basak Yanar

**Affiliations:** 1 Institute for Work & Health, Toronto, Ontario, Canada; 2 Dalla Lana School of Public Health, University of Toronto, Toronto, Ontario, Canada

**Keywords:** emergency responders, mental health, Ontario, social stigma, workplace

## Abstract

**Introduction:**

Mental health stigma has been a long-standing issue in public safety professions and can deter public safety personnel (PSP) from accessing mental health support. This is concerning as PSP experience higher rates of post-traumatic stress injuries (PTSI) than the general population. Public safety employers play an important role in stigma reduction. However, there is little research on Ontario public safety employers’ perspectives on mental health stigma in their organization and the accompanying organizational challenges they face in addressing stigma and supporting PSPs’ mental health.

**Methods:**

A thematic analysis of 28 semi-structured interviews with 33 public safety employer representatives from fire services, paramedics, police, and provincial corrections within Ontario was conducted.

**Results:**

Employer representatives recognized mental health stigma existed historically. They described that stigma is reducing due to sociopolitical changes that restructured PTSI as a common occupational injury. Participants shared ways they are adjusting organizational practices and policies to further reduce stigma and support PSP. They also highlighted accompanying organizational challenges, including ongoing historic stigma, uncertainty in how to support PSP with PTSI, and difficulty finding meaningful accommodations.

**Conclusion:**

Participants perceived mental health stigma to be decreasing in their public safety organizations. However, ongoing stigma, organizational factors and uncertainty around how to support those experiencing PTSI can pose challenges to return-to-work and accommodation. While in various stages of implementing initiatives to support mental health, organizations need to continue to build PTSI awareness, take accountability for their role in reducing mental health stigma, and build trauma-informed practices and policies.

**Highlights**

- The presumptive legislation and broader societal shifts in discussing mental health aided in reducing mental health stigma in public safety organizations.
- Employer organizations are in different stages of adjusting organizational practices and policies to further reduce stigma and support PSP.
- Organizational challenges, including ongoing historic stigma, uncertainty in how to support PSP with PTSI, and difficulty finding meaningful accommodations, are barriers to decreasing stigma.
- Organizations need to continue to build PTSI awareness, take accountability for their role in reducing mental health stigma, and build trauma-informed practices and policies.

## Introduction

Public safety personnel (PSP) are regularly exposed to potentially psychologically traumatic events in their work that can result in post-traumatic stress injuries (PTSI), such as post-traumatic stress disorder (PTSD), depression and anxiety.^1^-^4^ PSP generally includes any worker trained to protect public safety and security.^5^ In this paper, the term PSP includes individuals in firefighting, police, paramedic, and correctional services. A growing body of literature over the past two decades has integrated evidence for work-related exposure to traumatic events to develop prevalence estimates for PTSD among PSP. A survey of 5813 PSP in Canada reported that 44.5% of PSP screened positive for at least one mental disorder. In this sample, roughly 19.5% of municipal and provincial police, 29.1% of correctional workers, 13.5% of firefighters, and 24.5% of paramedics screened positive for PTSD.^6^ Another sample of 5267 PSP across Canada reported 22.1% experienced PTSD.^7^ For comparison, 8% of the general population in Canada experience mild to moderate symptoms of PTSD, with 5% reporting a formal diagnosis.^8^

Public safety organizations face multiple challenges in recognizing and accommodating PTSI among PSP. One challenge is the well-documented influence of stigma. Stigma is a social phenomenon that involves labelling a difference in identities or characteristics as important or relevant, holding negative beliefs or attitudes about this difference (i.e. stereotyping), and linguistically separating people with these differences from those without. There are often pre-existing power imbalances between the stigmatizer and those stigmatized, with the stigmatizer usually having more power due to some aspect of their identity.^9^,^10^ Stigma often leads to people (un)consciously treating others with the stigmatized identity differently, often negatively, which reinforces power imbalances.^9^,^10^ Hence, stigma is an active process of negatively defining and devaluing an identity or characteristic through actions and beliefs.^10^ It is multidimensional in that it occurs on an individual, organizational, and societal level.^10^ As a process, stigma can emerge, be maintained and can be removed.^10^,^11^

In the context of public safety organizations, there can be a predominant belief that individuals with mental health challenges are unable to cope with the stresses of their role and pose a safety risk.^12^ A meta-analysis of studies that explored PSPs’ experience of mental health stigma and related barriers to seeking support reported that roughly a third of PSP experience mental health stigma.^13^ Fear of judgment, unequal power dynamics and internalized beliefs of being incapable or flawed can lead to shame.^14^ This shame is exacerbated by fears of discrimination (i.e. being treated differently for having PTSI), disbelief (i.e. peers questioning the legitimacy of the PTSI), and loss of identity and professional status.^11^,^13^,^15^,^16^ In a survey of 133 police officers in Ontario, over half believed that in general, police officers perceive seeking mental health support as a personal failure and believed most officers would not work with a peer experiencing PTSI.^17^ These beliefs, if held by individual PSP, are documented barriers to seeking mental health support.^16^,^18^,^19^

Currently, PSP employer organizations use a variety of tools aimed to lower stigma and prevent mental health challenges, including peer support, critical incident stress management programs and psychoeducation on trauma and resilience through programs like Road to Mental Readiness and Before Occupational Stress. While these supports may lower the prevalence of some mental health conditions, these resources minimally change PSP willingness to access supports, such as a community mental health professional or their employee (and family) assistance program, which provides employees and their families with a range of free and confidential resources (such as counselling) offered by a third-party.^20^ In one study, 44.4% of police officers reported that they would not seek mental health supports if needed.^17^ Stigma-related barriers to seeking support primarily concerned service confidentiality, potential negative impact on their career, and fear of judgment from peers and leadership.^13^ Organizational culture is a contributing factor to the use of organizational supports, like the employee assistance program.^21^ Public safety employer organizations play an important role in addressing the causes and consequences of stigma related to mental health needs in their organization.^22^-^24^ Organizations may reinforce stigma structurally through practices and policies that disadvantage those with mental health conditions.^12^,^25^ Leadership within organizations may also hold negative beliefs about individuals with PTSI, which can result in discriminating actions and influence the general organizational culture and attitude towards mental health.^12^,^25^

***Ontario context***

In 2016, the Ontario Legislature amended legislation to designate PTSD experienced by PSP as an occupational injury. The legislative amendment, applying to the operational policies of the provincial workers’ compensation authority, presumes that a diagnosis of PTSD by a psychologist or psychiatrist for an active-duty PSP was caused by workplace exposures.^25^ This legislation covers 12 categories of workers, including police officers, paramedics, and workers in correctional institutions. One consequence of the presumptive legislation was a dramatic increase in the incidence of accepted workers’ compensation claims for PTSD diagnoses among the 70 000 PSP in Ontario, from fewer than 100 per year prior to 2016 to an average of 700 to 800 annual claims following the presumptive designation.^21^ In the case of work-related illness or injury compensated by the provincial workers’ compensation authority, employers have a legislated duty to accommodate workers with disabilities and provide meaningful opportunities to return to employment. In parallel with the legislative amendments, public safety organizations were required to provide the Ontario Ministry of Labour, Immigration, Training and Skills Development with a PTSD prevention plan that outlined what measures they were going to take to prevent the development of PTSD and support PSPs’ mental health.^25^

Public safety employers in Ontario also have the duty to accommodate members recovering from PTSI. If a PSP’s claim for a mental stress injury is approved, the Workplace Safety and Insurance Board (WSIB) supports the PSP while they are out of work and acts as a liaison between the employer, PSP, and the health care practitioner(s). PSP who experience PTSI tend to have longer claim durations and more difficulty returning to work for many reasons. Workers often struggle with a lack of communication from WSIB, financial ambiguity, and difficulty navigating the claim process.^26^ PSP can be reluctant to return-to-work (RTW) given their knowledge of the work demands and environment, which can activate trauma responses.^27^ Organizational factors such as staffing shortages, leadership’s lack of knowledge on PTSI, and lack of organizational support can leave PSP feeling unsupported by their employer, result in poorer mental health, and be a barrier to RTW.^28^,^29^ Stigma may also negatively impact the RTW journey of PSP who experienced PTSI.^30^ Despite the large influence public safety organizations have on PSP, little is known about employers’ perspectives on mental health stigma within their organizations. 

This paper describes the perspectives of representatives of public safety employers in Ontario concerning the historical influence of stigma, and the contemporary challenges arising from employer obligations to provide employment accommodations for PSP recovering from mental health injury attributed to PTSI. The study uses reflexive thematic analysis to explore how public safety employer representatives understand the historic and contemporary influence of mental health stigma within their organization.

## Methods

The findings come from data collected as part of a larger qualitative study that examined employer perspectives on RTW for PSP who experience PTSI. These findings are reflective of the rich discussions of mental health stigma that arose during these interviews. The broader study was reviewed and approved by the University of Toronto REB (RIS #3770). 

***Recruitment***

Employer participants were recruited through informal and formal referrals. For formal referrals, the senior representative with decision-making authority agreed to the participation of their organization; they were asked to nominate one or more members of the organization’s human resource or disability management staff as candidates to participate in the study. Informal referrals involved asking contacts known to the research team to circulate study information, identifying and contacting potential participants via their organization’s webpage or LinkedIn account, and a snowball sampling approach. Potential participants recruited from formal and informal referrals were told that participation is voluntary and confidential. 

***Participants***

Individuals who were employed by public safety organizations in Ontario, including police, correctional, paramedic, and fire services, were interviewed. Eligible participants had to have knowledge and experience with disability management and accommodation policies and practices, specifically relating to PTSI. Federally administered services were excluded.

Twenty-eight semi-structured interviews were conducted between July 2023 and January 2024 with 33 representatives (27 female, 6 male) from 28 public safety organizations spanning 16 municipalities, with five organizations servicing all of Ontario (Table 1). Public safety employer organizations included individual correctional institutions, and police, fire, and paramedic services that supported specific geographical areas. Participants were in various organizational departments including human resources, disability management and wellness, and leadership. Interviews were done either one-on-one with the interviewer or with pairs of participants, at the participants’ request. Purposive sampling was used to obtain a similar ratio of police, fire, corrections, and paramedic employers proportionate to their psychological injury claims reported to WSIB between 2018 and 2020. However, difficulty in recruiting eligible fire and corrections services led to less recruitment in these professions. 

**Table 1:** Number of participants per sector

Type of participant	Fire	Paramedic	Police	Corrections	Total
Employer organizations	4	9	11	4	28
Individual employer representatives	5^a^	10^b^	14	4	33

^a^ Two participants were involved in the return-to-work process for fire and paramedic. 

^b^ One participant was involved in the return-to-work process for paramedic and police. 

Most municipality-specific organizations were located Southern Ontario, with a minority in Northern Ontario. Municipalities sampled covered large, medium and small communities ranging from populations of over 1 million people to under 100 000 people. Nine organizations had over 1000 employees, five had between 500 to 1000 employees, and 14 organizations had under 500 employees. 

Interviews lasted 45 to 65 minutes (mean=53.39, SD=4.90) over Zoom (Version 5.13.5, Zoom Communications, Inc., San Jose, CA, USA) or phone and were recorded via a handheld recorder or through the Zoom platform. Verbal consent for participation and recordings was obtained at the beginning of interviews. Recordings were transcribed by a professional transcription service. LV de-identified and reviewed all interview transcripts for accuracy prior to analysis. The first two interviews were conducted by BY and LV to assess quality and flow of interview questions. The interview guide was adjusted accordingly. The remaining interviews were conducted by LV. BY, the co-principal investigator in this study, is a qualitative researcher with expertise in organizational behaviour and human resource management. LV, the research associate in the study, has qualitative research experience with a background in social anthropology and mental health treatment.

***Analysis***

Reflexive thematic analysis was used to engage in a rich and detailed analysis of the data by using the subjective knowledge and experience of the research team to co-create meaning within the data.^31^ Data were analyzed concurrently with interviews, which helped identify gaps in knowledge, inform subsequent interview questions, and identify themes early on. The research team stopped conducting interviews when there were enough data to address the research question.^32^

LV and BY familiarized themselves with the data and met regularly to discuss interpretations of the data, which resulted in them inductively creating codes to label key reflections.^33^ The first few interviews were coded individually in NVivo10 for Windows (QSR International Pty Ltd. 1999–2014, Burlington, MA, USA) by LV and BY to help with data familiarization and interpretation. Afterwards, LV and BY discussed their interpretations and co-created codes that were applied to all transcripts. LV did the initial interview coding and highlighted her additional or changing interpretations. BY reviewed the coding and reflections and included her own thoughts. These curiosities were discussed in team meetings, and codes were adjusted accordingly to highlight these new meanings. Codes were synthesized to create larger descriptive patterns of meaning in the data shared by the research team (i.e. themes). Themes were continuously refined throughout analysis until they were succinct and distinct from each other. CM participated in co-creating meaning by providing his interpretations on the broader themes. Coding and theme creation occurred between November 2023 and May 2024. 

Weekly team meetings were held between all team members throughout the study, which allowed for ongoing discussions about recruitment, methods, and interpretations. Reflexivity was maintained throughout by discussing in team meetings their curiosities as situated in their interests and the connections they were making between their area of expertise and the data.^34^ They also engaged in reflexivity by reflecting on and discussing the underlying assumptions they were drawing from when analyzing data, how these assumptions influenced the analysis and how the interpretations would impact PSP, WSIB, and employer organizations. This was particularly helpful for moments when participants shared strong opinions that had the potential to overshadow the nuances of the situation described by other participants. Furthermore, BY and LV’s academic backgrounds and interests helped attune them to the nuances in participants’ conversations about stigma in their workplace and profession and situate it within a larger cultural and political context. An audit trail also supported reflexivity by tracking team members’ questions, evolving interpretations of the data, and changes to the research process. A summary of the research findings from the broader component this study was based on was provided to select participants from all PSP professions for feedback to further enrich the data by involving them in the research process and data interpretation. The Standards for Reporting Qualitative Research were used to guide the reporting of this study.^35^

## Results

**Figure 1:**
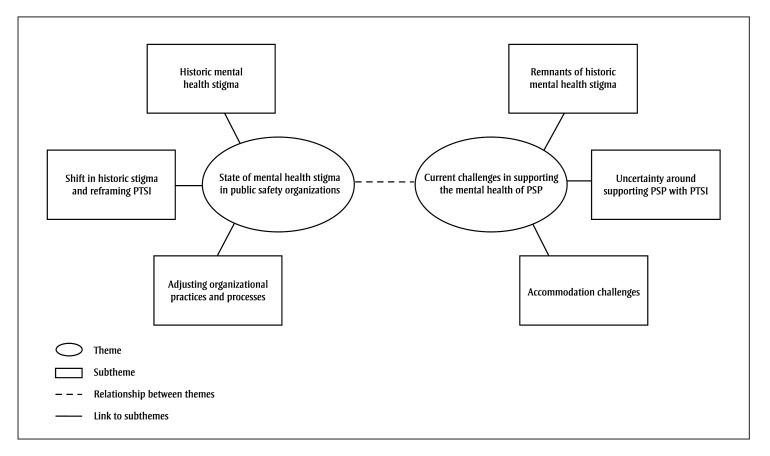
Thematic map with themes, subthemes and relationships between themes

**Abbreviations: **PSP, public safety personnel; PTSI, post-traumatic stress injuries. 

Participants explained that historic mental health stigma, which positioned PTSI as an inherent shortcoming and a sign that a PSP is unfit for the role, is slowly diminishing throughout public safety organizations. They attributed this shift to broader societal changes that embrace mental health and legislative reforms that defined PTSI as an occupational injury and require employers to provide employment accommodations. This reframing encouraged public safety organizations to re-evaluate their practices and create and strengthen mental health initiatives and supports to reduce mental health stigma and improve the prevention of PTSI. Participants also discussed challenges related to supporting the mental health of PSP, which may impact the effectiveness of the efforts to reduce stigma (Figure 1). These challenges will be addressed in the next sections.

***The state of mental health stigma in public safety organizations***

**Historic mental health stigma**

Employer representatives explained that historically there were substantial stigmatizing beliefs in their organization surrounding mental health. PSP were expected to remain in control of their emotions during highly stressful events and not express vulnerable feelings, such as fear and sadness. There was a belief that “Yeah, this is your job, this is what you signed up for.” (paramedic, interview 19) PSP who expressed struggling with their mental health were morally stigmatized as it was assumed that they were inherently emotionally weak and were not capable of being in the profession. There was also the all-or-nothing assumption that PSP are either fully capable of doing their job or completely incapable. A corrections employer representative shared how when they were discussing exposure therapy, there was a lot of resistance from supervisors due to their lack of knowledge in this approach and their belief that “you either come back to work, or you don’t” (interview 29). These negative attitudes concerning mental health also fed into PSPs’ concern of discrimination by their employer. Specifically, participants described that PSP feared that their career would be jeopardized as they may not be considered for promotions or may be forced to stop practising in their current professional capacity. A police employer representative highlighted, “I remember officers sitting in my office afraid to speak up because they think their gun will be taken away” (interview 33). Another police employer explained that concerns of being stigmatized were “reinforced by experiences,” which influenced “why people choose to do things in a certain way” (interview 6).

PSP often found it difficult to accept the impact of the job on their mental health as they were often fearful of stigma and discrimination or felt ashamed of their condition. A paramedic employer representative explained, “Nobody would admit a mental health injury before. They’d go off on anything except mental health. A back injury, or a shoulder strain, or whatever” (interview 21). Difficulty accepting their mental health challenges and needs also deterred PSP from seeking support from mental health professionals, as another paramedic employer shared: 

It was very much the suck it up attitude and move on, and nobody talked about going and talking to a psychologist. Like, what was a psychologist. That was never a conversation. (interview 22) 

Participants shared that denying feelings that arose from the job often led to increasing mental distress or using harmful methods to manage the emotions themselves, which could involve numbing practices such as substance use. A police employer described how drinking was encouraged in their policing culture as a coping mechanism: 

When [police officers] went to Police College, they were taught if you had a bad day, you had a stressful day, you take your guys out, you go to the bar, and you get drunk. You get up the next morning and you just deal with it. (interview 11) 

Alternatively, a few participants described approaches to avoid stigma by seeking support from trusted colleagues and informally debriefing with them after a potentially traumatic event. 

**Shift in historic stigma and reframing PTSI**

While participants recognized historic stigma continues to exist, they also recognized that it is diminishing in their organization. They attributed this change to two main factors—broader social shifts in understanding mental health and the introduction of legislative reforms in 2016. These social and policy shifts helped to normalize discussions of mental health and reframed PTSI as an occupational injury, which encouraged organizations to bolster preventative mental health supports for their members and improve accommodation and RTW practices. 

Employer representatives discussed how the public’s relationship with mental health changed over the years. They noted that society is more open to discussing mental health, which is evident from content shown in mainstream media. A paramedic representative described the shift towards mental health awareness in the media: “Twenty years ago, you wouldn’t have seen the ads that you see now talking about mental health and the programs, and if you are feeling overwhelmed, you need to talk about it” (interview 7). Participants suggested that society’s focus on mental health helps reduce stigma among PSP, specifically among the more recent generation of PSP who have been more exposed to messages normalizing mental health. Participants shared that this generational shift is evident in how newer PSP tend to be more vocal about their mental health status and needs and take a more active role in advocating for their mental health. A police representative observed how media has changed generational beliefs:

There is more social media and campaigns publicly on the whole mental health aspect, you can definitely see it in the younger recruits. They’re more willing to come forward and more willing to talk about themselves and just advocate for themselves and for more wellness supports. So, that’s the shift where the older generation may not necessarily be there, but definitely all our new recruits coming in are just more knowledgeable and more in tune as to what they’re willing or wanting to do. (interview 3) 

Given this normalization of mental health on a societal level, some participants described that the newer generation of PSP tend to be more self-aware of their emotional state and engaged with mental health supports. Some participants described that the influx of PSP who do not hold mental health stigma is changing the broader organizational and professional culture as older PSP who hold stigma are retiring. As one police employer representative described, “there is a bit of an old guard that is passing through our service.” (interview 6)

However, this openness to discussing mental health challenges and seeking help is generally spreading across different generations of PSP across all services. A corrections representative noticed PSP are more “transparent” about mental health challenges and “are speaking up about [having a] mental illness and [how] they’ve been handling it” (interview 16). In addition to discussing the resources PSP use to manage PTSI, PSP can also be eager to make use of professional supports available. At a fire services employer organization, a staff psychologist who initially worked with peer supporters expanded their services to the whole organization after receiving an overwhelming interest from PSP. Now, over three quarters of the organization see the psychologist. Similarly, a police employer recalled a moment where a PSP openly acknowledged accessing the employee assistance program during a training session and praised those services, which the participant noted would not have been discussed before.

Additionally, presumptive legislation and the requirement to create a prevention plan actively placed responsibility for PSP wellness onto the public safety organization and encouraged the leaders, managers and coordinators of the organization to reflect and act on how to adequately support their employees’ mental health. This refocusing was highlighted by a fire services employer representative who acknowledged that the presumptive legislation “definitely put the focus on mental health in the workplace. It made us focus on ‘we’ve got to ensure that everything we do is to prevent these claims from even happening’, so a lot of prevention” (interview 1). Since the presumptive legislation, participants have also noted an increase in PTSI claims, and more PSP reporting and documenting stressful events with their employer. For some, the increase in claims resulted in creating or expanding abilities management teams who support claims and RTW. For one police employer, the increase in claims resulted in contracting a third-party disability management service provider until they could expand their internal team. 

The shifts in understanding mental health and the legislative change were reflected in participants’ discussion of PTSI. Whereas historic stigma represented PTSI as a personal failing, participants explicitly spoke of PTSI as an occupational hazard. This shift in language was evident to one paramedic employer:

I don’t think it would have been strange even 10 years ago to say, [a PSP] has gone off sick, he’s loopy, or crazy, or anything like that. There might still be that stigma, but I think at least the language has started to change. They’re off with a mental health injury, or a stress injury, or something like that, or even PTSD or whatever. I think people are more inclined to at least use the vernacular that we’ve become accustomed to. And I think if you [ask] when did we probably start seeing that change, I think it was probably shortly after the presumptive legislation for PTSD came into play for paramedics. (interview 21) 

This reconstruction of PTSI as an unavoidable consequence of work exposures also came through subtle nuances in the way employer representatives described mental health in the public safety profession. They defined PTSI as a complex injury whose impact fluctuates over time and can present differently in each person, which requires organizational initiatives that focus on supporting PSP who experience PTSI: 

[PTSI is] not the same thing as you break a wrist, I break a wrist, and we’re suffering the same thing. It is not the same as a mental health thing. If you’re suffering from PTSI and I’m suffering from PTSI, we’re such unique individuals in our brains. Something that might trigger me won’t trigger you, as an example. This leads to why we need to invest more resources in supporting those employees because you just don’t know what you’re going to get. (paramedic employer, interview 17) 

**Adjusting organizational practices and processes**

Participants described that the broader social shifts and the presumptive legislation encouraged public safety organizations to create and strengthen mental health initiatives and supports to reduce mental health stigma and improve PTSI prevention. Given PTSI was positioned as a common occupational injury, organizations needed to educate PSP, supervisors, and leadership on PTSI and mental health in their organization. There was also a need for more proactive supports that prevented the onset and development of PTSI. Hence, organizational practices and processes needed to change to suit the new understanding of PTSI. 

The supports described by participants involved training for PSP and supervisors to create a baseline understanding of mental health and build awareness of PTSI symptoms in themselves and others. Participants listed using online training courses, such as Road to Mental Readiness and Before Operational Stress, and a few noted contracting a local mental health professional to provide training. Some supervisors were given specialized training primarily by internal wellness or claims management teams on how to support PSP with PTSI, understanding cognitive limitations and restrictions, their duty to accommodate, and how to identify appropriate accommodations. A police employer described a yearly discussion they have with leadership: 

[On the education day] every year, and we have had time with every single one of them that has come through to talk about abilities management, return to work, our role, their role. So, sometimes it is a 10-minute conversation, there are no questions. We’re like, okay, well, you got the information, you know who we are. And other times I have had these wonderful hour, hour-and-a- half long conversations with people about how we can do better, and their pain points. So, just really rich ideas, and open, honest conversations about, why do we do it like this, and we need to do this, and why don’t we do that? (interview 32) 

Some employer representatives described the role of leadership in normalizing mental health discussions in their organizations. A few shared ways that their leadership team is challenging stigma through creating an organizational strategic mission to support PSPs’ wellness and implementing more organizational mental health supports. A police employer representative shared how leadership’s verbal encouragement of accessing mental health supports helped lower stigma: 

Our command staff, they very much have the attitude of no, I want you to be accessing these supports. It’s not a sign of weakness, it’s a sign of strength so it’s that we’re trying to shift the culture from the top down, but as you know, sometimes it’s not as quick, but I think we’re getting there. It will take a lot of time to undo the years of that behaviour, right? (interview 11) 

Other initiatives included having an on-site psychologist, implementing peer support teams, critical event debriefs, and establishing wellness teams with individuals specializing in mental health and disability management. A few participants described having committees where employer representatives and union representatives meet to discuss how to support PSP mental health. Participants who had these committees in their organizations shared that they were useful for sharing knowledge about PSPs’ experience and keeping each other accountable: 

We do have a program in place [for] whenever there’s an accommodation that needs to be implemented. So, the union has buy-in, the manager, the union member, the employer and another [program] representative which is usually another union member, sit together collaboratively and they figure out what accommodations are going to work best. I think the union is very helpful when it comes to that aspect, only because by nature if management might suggest something, there may be some pushback, but if the employee sees the union and management are in agreement with the accommodation plan, then there’s not as much resistance to it. (corrections employer, interview 37) 

While public safety organizations have taken steps to strengthen their policies and practices to align with the understanding of PTSI as an occupational injury, participants also discussed challenges related to supporting the mental health of PSP. These challenges may impact the effectiveness of their efforts to reduce stigma. 

***Current challenges in supporting the mental health of PSP***

**Remnants of historic mental health stigma**

Employer representatives acknowledged that the experiences that arose from historic stigma continue to shape organizations’ relationship with PSP. While the staff, beliefs, and structure of the organization may have changed to one that is more supportive of mental health, many PSP struggle to trust in this change: 

Even though we are different people, and our approach may be different than in the past, if somebody had a terrible experience in the past and has a bad taste in their mouth from 15 or 20 years ago, we have to overcome that first, and then deal with the issue of the day. So, some of the resistance or challenge we get might not actually be about us, it’s about what happened a decade or more ago. (police employer, interview 32) 

For some, this mistrust was warranted as there continues to be suspicion towards PSP experiencing PTSI, wondering if the person is being dishonest about their injury to receive paid time off. A police employer described how this suspicion can be born from inaccurate beliefs of what a mental health occupational injury looks like: 

So, I think what we need to recognize in policing is that it’s a suspicious group who are trained investigators, so they fill in the blanks. And I think sometimes that can be to our detriment from a cultural perspective in terms of oh well, I saw this person in the gym, or I saw them on Instagram, and I don’t know why they’re not here because they look really good and they’re travelling or doing whatever. The rumour mill is brutal. (interview 32) 

Employer representatives described that having management educated on mental health and on board with mental health advocacy is critical to fostering a supportive workplace culture and challenging stigma, which helps (re)build trust with employees. Participants shared their efforts in educating and offering guidance to management on PTSI claims, and mental health awareness and advocacy: 

So, we’re trying to provide more mental health training. The psychologist I said we work with; we’re arranging for her to come back. She was in a couple of years ago, but [this time] she’s going to have sessions with everybody to talk about stigma, recognizing signs and symptoms, and just different things, and also, specific training for supervisors on how to handle when someone has had a traumatic call, but also, how to handle the people that are left afterwards. Explaining to them where this [affected] person is, or if they are saying things they shouldn’t say, so how to just handle that in the workplace. Hopefully, that will help. (police and paramedic employer, interview 16) 

**Uncertainty around supporting PSP with PTSI**

Participants shared that while there was more acceptance and acknowledgement of mental challenges within their organizations, supervisors and colleagues who were adapting to the changing understanding of PTSI as a complex and individualized injury expressed uncertainty with how to communicate and support these workers. Most colleagues and supervisors generally accept- ed the legitimacy of PTSI, however, they were “afraid to talk to [PSP experiencing PTSI] because [the colleague] might set them off” (police employer, interview 28). As described by a paramedic and fire employer, this can leave supervisors and colleagues hyper-focused on the condition and how their interactions with the PSP can impact the person:

Everyone is so scared of this topic [of PTSI]. They’re scared of certain things they say. Everything is toned down and everything is monotone. Let’s just say you and I are dealing with someone and you are the WSIB rep, and we’re having this conversation with an employee. We end the meeting and you’re a little harsh. And the employee ends up doing something to themselves, inflicting harm on themselves. Now there’s that sense of ownership that I caused [the harm]. That’s what I mean by scared because we don’t see [the PTSI], we don’t feel it, we don’t know hardly anything about it, because that’s owned within somebody’s system. I think it’s just people are afraid and they don’t really understand it. (interview 8) 

For some, there was a shift from believing PTSI is an inherent sign of one’s weakness to a belief that mental health is influenced by many factors, such as what is said to them, and those struggling with their mental health are extra sensitive to these factors. This shift in stigma can have similar impacts to historic stigma as it isolates the PSP by othering them. It frames PTSI as central to the PSP’s identity, forgetting the other aspects that make them a PSP and an individual, thereby reducing the PSP’s personhood to their experience of PTSI. 

Organizational uncertainty in understanding PSPs’ needs and possible accommodation placements also made it difficult for organizations to create policies and procedures that provided guidance on how to relate with PSP who experience PTSI. For some supervisors, this uncertainty came from vague limitations and restrictions from WSIB and health care providers, which created challenges around supporting the RTW and accommodation of the PSP: 

Sometimes we get a lot of, can’t be exposed to traumatic content. So, I need that fleshed out a little bit. What does that mean? Does that mean traumatic content in the context of an investigation? Does that mean traumatic content on TV, because we have [the news] on in this building all the time? So, I think really fleshing out what our jobs require and what people require is where we need to start, and then we can start matching. But, like I said, that information right now isn’t there. (police employer, interview 2) 

Employer organizations shared that PSP experiencing PTSI are often restricted from cues and duties that are critical to their pre-injury position. These cues can be present in the physical facility, making it difficult to accommodate. Employers expressed the importance of PSP having multiple avenues to access supports that can address their various needs. Participants described various initiatives to bolster PSP support systems, such as having peer support as part of the standard RTW process. Participants also mentioned that building trust between the employer organization and the PSP helps facilitate a successful return as PSP are more transparent about their needs as they move through the RTW plan. This transparency gives employer organizations the opportunity to step in and work with PSP to have their needs met.

Participants also discussed gradual RTW processes that worked closely with PSP to tailor the plan to their needs. For some, PSP were offered the opportunity to do exposure therapy in the workplace and have a dedicated support person throughout the RTW journey. RTW plans were evaluated regularly with the PSP to ensure the appropriateness of pace, work, and support.

**Accommodation challenges**

Employer organizations recognized that PSP value and pride themselves on their professional identity. For many PSP, their job is an important aspect of their identity and lifestyle. They explained that if PSP are accommodated in roles where the key functions of their identity are not included, they may not feel that the work is meaningful. Given the importance of their professional identity, returning to work with accommodations may be seen “as a downgrade” (fire employer, interview 1).

I think the thing that is probably the most impactful is the self-stigma. There’s that self-perception, and sort of self-degradation of, I’m not the person I used to be, other people are going to think that as well. That reduces my value to the service, it reduces my credibility as a member of the service, I’m going to be seen as not capable of doing what I ought to be doing, having failed the service, having failed the public. (police employer, interview 6) 

Furthermore, employer representatives shared that the severity of PSPs’ restrictions and limitations and the specialization of their work can make it hard to pair PSP with meaningful work. This was especially true for smaller organizations with limited resources and job openings, and corrections and paramedic services where there is less diversity of roles available that do not involve exposure to potentially traumatic events or their functional restrictions. A paramedic employer described their limited roles available within their service: 

Each base has a base manager, an assistant, and then the only other people at that base are the frontline staff. It’s not like I have a book that I can go, okay, yes, you have these restrictions, you can do this, this, this and this, because we don’t have that availability. (interview 14) 

Difficulty in accommodating PSP can lead to many individuals being accommodated in a specific role or department, which can be seen as a career-limiting move. For some, suitable accommodations are roles held by senior officers, which can create friction as described by a corrections representative:

[When a correctional officer] can’t work their post, well, you pull them out of that post and then [the accommodated person is] taking a position from a senior officer. Seniority is very, very important. Your seniority gives you your post picks, and a lot of times post picks, it’s the preferential schedule, those types of things, and that’s what you earn with your seniority. However, a lot of those posts are good accommodation posts as well. So, that’s where some of that frustration is. (interview 29) 

Compounding accommodation challenges is the stigma supervisors may hold around accommodated work, which can make it difficult for PSP to RTW and reintegrate into the employer organization. Supervisors who stigmatize accommodations may believe that the PSP is not fit to return, drawing from the all-or-nothing assumption mentioned earlier. This can limit supervisors’ participation in accommodating PSP as they may be reluctant to accept an accommodated worker: 

I think even sometimes internally the challenge is getting supervisors onboard to have accommodated members within their units. I think there is a lot of stigma out there for it, so just kind of breaking those barriers, right, that even though they have these restrictions, she’ll be able to do this, this, and this, and this, which will help ease the workload of the other members and then they can do. So, I think it’s just sometimes showing them a different perspective of it. (police employer, interview 33) 

As this participant shared, educating supervisors on identifying appropriate accommodations that utilize the PSPs’ skills helps challenge accommodation stigma. Other supervisory education described by participants included the duty to accommodate and understanding cognitive restrictions and limitations. A participant went on to explain that “[The] whole education piece is sometimes helpful so [supervisors] understand the importance to the member and also to the service in terms of the responsibilities that we have to look at modified work” (interview 33).

Generally, participants described the importance of being creative in identifying accommodations that consider the organizational needs and the skills and limitations of the PSP. This creativity took different forms, including finding accommodations that align with the PSPs’ hobbies, previous education or work experience, and breaking down positions into tasks that are bundled as an accommodation. For many public safety employers who were administratively integrated into their municipality, creativity involved finding positions in different departments of the city. 

We have different aspects of modified work that doesn’t involve you needing to do any paramedicine. [PSP] are most successful when we’re looking outside of paramedicine completely. And being [part of the municipality], we have [many divisions]. It’s proving to be better where they’re completely removed out of paramedicine and into a different role while they’re recovering. (paramedic employer, interview 35) 

A couple of participants mentioned the benefit of framing work as a part of the treatment process to fuel PSPs’ encouragement to RTW. While a few participants explicitly named this, many members acknowledged the “better at work” principle36 and regularly stated that the longer someone is off work, the harder it is for them to return. This reframing placed importance on gradually exposing PSP to the workforce and building their confidence in returning. 

We need to understand the times where [PSP are] able to do something and we can work with them to do a search and see if there’s something temporary available for them, just to maybe test the waters for them as part of their treatment. We need to understand what their functional abilities are, their limitations, so that we can put things in place for them and support their return to work. (fire employer, interview 1) 

## Discussion

PSP in fire, police, paramedicine, and correctional service occupations have high exposure to potentially psychologically traumatic events in their work that can lead to PTSI. A large barrier to seeking treatment and RTW is stigma, which is the negative perceptions of specific characteristics that can lead to the person being treated unjustly. In this paper, we described the perspectives of public safety employer representatives on the historical influence of stigma, and the current challenges tied to employer obligations to accommodate PSP who experienced PTSI. 

Generally, employer representatives recognized that mental health stigma is decreasing among PSP as PTSI is becoming more accepted as a common occupational injury. This change was primarily attributed to broader societal changes that supported the recognition of the importance of mental health and legislative reforms that defined PTSI among PSP as an occupational injury. Arising from the legislative reforms, participants recognized the obligation on employers to accommodate PSP recovering from PTSI and spoke positively of organizational initiatives to strengthen PTSI prevention efforts and decrease the influence of mental health stigma. This reframing encouraged public safety organizations to innovate in creating and strengthening mental health initiatives and supports to reduce mental health stigma and improve prevention of PTSI. 

Participants also spoke of challenges related to supporting the mental health of PSP. There was broad acknowledgement that the remnants of historic stigma are present in organizations. Additionally, however, representatives spoke of challenges arising from uncertainty about how organizations can best support the recovery and accommodation of PSP disabled by PTSI. Some organizations have limited options for suitable accommodations, and representatives noted PSP and supervisors may hold stigma towards accommodated employment.

Organizations in our study were at different stages of developing shared knowledge, awareness, and values around mental health and creating a shared vision for their organization’s future that prioritizes PSP mental health among peers, staff, and leadership. Building common knowledge, values and vision has the potential to reduce stigma within PSP, between peers, and among leadership.^30^ This requires shifting the organization’s fundamental approach to mental health. One approach is creating a trauma-informed workplace, which is effective in building trust and reducing stigma.^37^ Being trauma-informed means recognizing that most people have experienced or will experience some form of trauma in one or more aspects of their life that change the way they perceive and interact with the world. It acknowledges the broader circumstances that influence someone’s behaviour and looks to understand what happened to the person rather than what is “wrong” with the person, being sensitive to the fact people are impacted differently by traumatic exposures.^37^,^38^ Trauma-informed practices aim to prevent traumatic instances, build resilience to adversities, and support those healing from trauma.^38^ Trauma-informed workplaces value and develop organizationally integrated practices that enhance psychological safety, transparency, empowerment, collaboration, and demonstrate a commitment to creating an inclusive and equitable environment. Psychological safety is an employee’s comfort in expressing their struggles at work (including mental health challenges), making mistakes, and disagreeing with colleagues or leadership.^37^ Generally, employers can openly acknowledge stigma in the organization and actively collaborate with staff through ongoing discussions and feedback to create initiatives unique to their organizational context that support mental health and create a culture of safety.^23^,^39^,^40^ While the general principles of being trauma-informed can help ease stigma and improve mental health, these practices need to be tailored to suit the unique culture and context of the organization. For example, organizations can create a common understanding of mental health through providing tailored and ongoing mental health training in the context of the public safety profession to all levels of the organization.^41^ Evidence supports that tailored training can help PSP recognize signs of PTSI within themselves^42^,^43^ and lower stigma towards others.^44^ Furthermore, integrating a variety of mental health supports within the organization, such as an in-house psychologist, peer support, or a list of mental health professionals in the community who are versed in the profession, can help lower stigma through improving access to supports, acknowledging the connection between work and PTSI, and empowering PSP to choose a support best suited for them.^45^ This aligned with many of the practices and supports shared by participants. Additionally, organizations can lower stigma and support mental health through consistent supervisor support via non-judgmental check-ins, and regular recognition and praise for the work of PSP.^38^ Furthermore, organizations can build a trauma-informed workplace by hiring additional staff to allow for better work/life balance, thereby diminishing the guilt and fear of having their mental health and integrity questioned by taking time off work or not working overtime.^46^ It is important for organizations to recognize that creating a trauma‑informed workplace is an ongoing process that requires a continuous commitment to tailor and adapt practices to ensure employees are empowered and safe.^38^

Tracking the effectiveness of initiatives and formalizing successful initiatives into policy makes sure that trauma-informed values and understandings of mental health are sown into the fabric of the organization, which can inform future practices and policies.^23^,^40^ At an industry level, broader initiatives that target resources, relationships, and legislation can help with longer term destigmatization efforts.^24^ For example, Szymanski and Hall 47 noted the potential value of mechanisms to audit public safety organizations’ PTSD prevention plans that they were required to create during the presumptive legislation. This could help address stigma on the industry level by holding organizations accountable to their mental health supports.

The findings of this study reinforce observations from previous research documenting the distinct features of stigma surrounding mental health disorders among PSP.^48^ This literature, based on studies across the range of public safety sectors (fire, police, paramedics, and corrections), consistently finds that PSP perceive colleagues and their organizations to hold negative attitudes and beliefs about occupational fitness among PSP with PTSI-related mental health conditions and that these perceptions can be a barrier to individual PSP seeking care. As a complement to this previous research, our study focused on perspectives of employer representatives with occupational roles in delivering disability management services in their organizations. This different vantage point reinforces observations from previous research while providing fresh perspectives on organizational strategies to mitigate or diminish the influence of mental health stigma. Participants in this study were also able to offer perspectives on the extent to which mental health stigma has changed over time within their organizations. Monitoring changing perceptions of mental health stigma among PSP is an important research opportunity for the future.

***Strengths and limitations***

A strength of this study is that the participants were from various public safety professions, which offered a broader perspective of stigma within the public safety field. Additionally, the unique position of participants as employer representatives who oversaw organizational PSP claims and wellness provided unique insights into the mental health stigma among PSP and organizational practices. These roles gave participants knowledge in the challenges, current practices, and aspirations of the organization relating to mental health support. 

This study has several limitations. As noted earlier, participants attributed some shifts in mental health stigma to the presumptive legislation in Ontario given that it legally legitimized occupational PTSD and held organizations accountable to PTSI prevention. Not all provinces and territories within Canada have a similar legislation, which may influence mental health stigma within public safety organizations in these areas. Hence, the transferability of these findings may be limited. Furthermore, this study focused on employer representatives’ understandings of stigma within their organization. This research did not explore mental health stigma within other stakeholders, such as PSP, supervisors, and WSIB. Understanding how stigma exists within these stakeholders can offer a more fulsome picture of how stigma is actively being maintained and challenged, and how it is transferring between stakeholders. Lastly, the data from this study emerged from research that primarily focused on employer’s perspectives of the RTW process for PSP who experience PTSI. Future lines of inquiry could also explore employer representatives’ relationship with mental health stigma, specifically asking how employer representatives understand their role in challenging stigma, and how they are trying to challenge stigma within themselves. It could also be useful to explore employer representatives’ reflections of the influence of various policies, practices and procedures on mental health stigma in their workplace.

## Conclusion

Generally, public safety employer representatives in Ontario shared that mental health stigma is decreasing in their organizations due to shifting understandings of PTSI as a common occupational injury. This change was in part due to the presumptive legislation and broader societal shifts. Despite this, organizational challenges can pose barriers for further stigma reduction. Employer organizations are in different stages of adjusting organizational practices and policies to further reduce stigma and support PSP. The current study provides unique perspectives on organizational strategies to decrease the influence of mental health stigma.

## Acknowledgements

This Project was funded by a grant provided by the Workplace Safety and Insurance Board (Ontario). The provision of grant support by the WSIB does not in any way infer or imply endorsement of the content by the WSIB.

## Conflicts of interest

There are no conflicts of interest. 

## Authors’ contributions and statement

LV: Data curation, formal analysis, investigation, methodology, project administration, resources, validation, visualization, writing—original draft. 

CM: Conceptualization, funding acquisition, methodology, supervision, writing—review and editing. 

BY: Conceptualization, data curation, formal analysis, funding acquisition, methodology, project administration, supervision, validation, writing—review and editing. 

The content and views expressed in this article are those of the authors and do not necessarily reflect those of the Government of Canada.
